# Pure white cell aplasia before and after thymectomy in the rare conundrum of thymoma: A case report and review of the literature

**DOI:** 10.1097/MD.0000000000036920

**Published:** 2024-01-19

**Authors:** Yang Yang, Chunmei Chen, Bingrong Zheng, Liping Fan, Xiajun Chen, Meiwei Hu

**Affiliations:** a Department of Hematology, the Second Affiliated Hospital of Zhejiang Chinese Medical University, Hangzhou, Zhejiang, China.

**Keywords:** cyclosporine A, pure white cell aplasia, remission, thymectomy, thymoma

## Abstract

**Rationale:**

Pure white cell aplasia (PWCA) is a rare paraneoplastic syndrome that occurs in patients with thymomas. Currently, the pathogenesis and treatment of this disease remain in the exploratory stage.

**Patient concerns:**

We report a 68-year-old woman with thymoma experienced PWCA involvement as her first presentation. The patient had high fever and agranulocytosis at the onset of the disease. The white blood cell count in the complete blood count was 1.9 × 10^9^/L with a neutrophil of 0.1 × 10^9^/L. The bone marrow aspirates showed decreased granulocyte proliferation. Computed tomography showed a large mass in the anterior mediastinum.

**Diagnoses:**

The final diagnosis of our patient was PWCA and thymoma.

**Interventions:**

She underwent a thymectomy and cyclosporine A administration during first remission.

**Outcomes:**

Long-term remission was achieved following the readministration of cyclosporine A after the disease recurrence.

**Lessons:**

PWCA or agranulocytosis with thymoma has been confirmed to be an extremely rare disease. Thymomas with PWCA correlate with autoimmunity. From this case study and the literature review, we concluded that the pathogenesis of thymomas in PWCA is mainly related to the activation of autoreactive T cells. Thymectomy and the immunosuppressive drug, cyclosporine A, were chosen for treatment. The patient’s granulocyte levels were unable to recover after surgery because of the inability to promptly clear activated T cells. After surgery, cyclosporine A continued to take for a long time. Thymectomy combined with prolonged cyclosporine A administration may be an effective method for treating this rare disease.

## 1. Introduction

Pure white cell aplasia (PWCA) is a rare form of neutropenia caused by the loss of the neutrophil lineage. It is characterized by the absence of granulogenesis, but results in normal production and development of erythroid and megakaryocytes in the bone marrow.^[1]^ From a clinical perspective, PWCA often results in repeated severe infections and even death due to sepsis. PWCA may correlate with thymomas,^[2]^ autoimmune diseases,^[3]^ drugs,^[4]^ or even idiopathic causes.^[5]^ PWCA is considered an autoimmune disease, and its immunological pathogenesis has been confirmed in some cases.^[6]^ There have been only few reports in the literature on cases of thymoma correlated with PWCA because of the rare nature of this disease; furthermore, the precise mechanism of the disease has not been elucidated.

Thymoma is a rare disease, accounting for over 20% of the total incidence of mediastinal tumors^[7]^ and has generally potential malignant features. It correlates with a wide variety of immune-mediated paraneoplastic syndromes (e.g., myasthenia gravis, pure red cell aplastic anemia, Good syndrome, and systemic lupus erythematosus). In contrast, PWCA is a rare paraneoplastic syndrome of thymomas. The treatment of thymoma with PWCA has not been established; however, various treatments have been attempted, including corticosteroids, cyclosporine A (CSA), cyclophosphamide, alemtuzumab, granulocyte colony-stimulating factor (G-CSF), high-dose intravenous immunoglobulin G, or plasmapheresis. Surgical removal of thymoma can also aid in the treatment of PWCA.

This study reports the case of a patient with primary agranulocytosis, who underwent bone marrow examination and was subsequently diagnosed with PWCA. Further investigation through computed tomography and biopsy revealed the presence of a thymoma. Agranulocytosis was observed before and after surgery. Granulocytes returned to normal after treatment with CSA combined with thymectomy.

## 2. Case presentation

A 68-year-old woman with “Hashimoto thyroiditis” experienced chest pain for 3 days. Laboratory tests conducted at the local hospital showed severe neutropenia with a white blood count (WBC) of 1.9 × 10^9^/L and a neutrophil (NE) of 0.1 × 10^9^/L. As a result, she was immediately transferred to our hospital.

**Figure 1.**
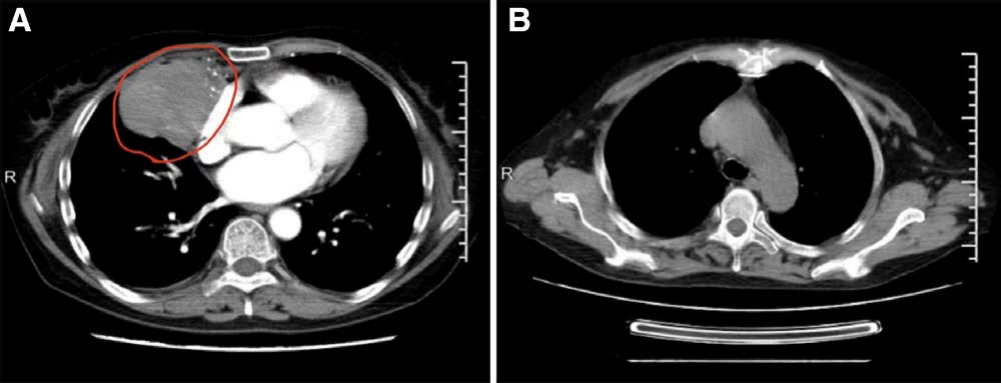
Computed tomography (CT) images of the patient. (A) CT showing an anterior mediastinal mass (7.6 × 6.3 cm). (B) Anterior mediastinal mass disappeared after thymectomy.

**Figure 2.**
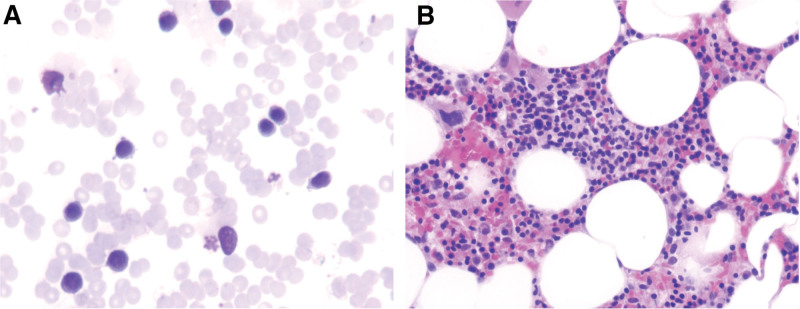
Routine and biopsy of the patient’s bone marrow. (A) Wright–Giemsa stain 400×. Bone marrow aspirate showing lymphocytes, erythrocytes, metablasts, and plasma cells with absence of granulopoiesis. (B) Hematoxylin and eosin staining 200×. Bone marrow core biopsy shows complete absence of granulocytic precursor elements.

**Figure 3.**
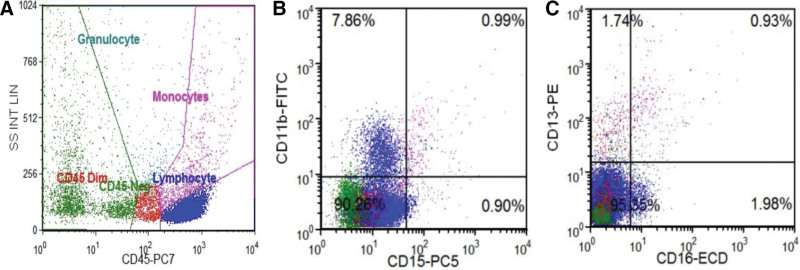
Bone morrow flow cytometry. (A) Grouping the collected cells using CD45-SSC showed a significant decrease in granulocytes, accounting for only 1.4%. (B, C) Granular expression associated antigens CD13, CD16, CD11b, and CD15.

**Figure 4.**
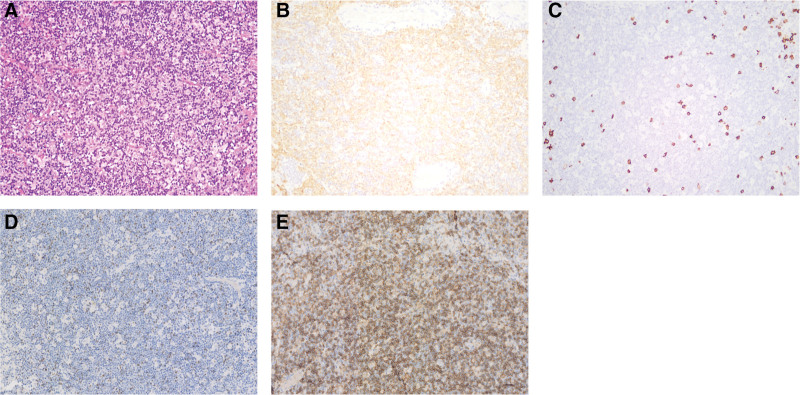
Pathology of thymoma in the patient. (A) Hematoxylin–eosin, 200×. The tumor tissue showing a large number of diffuse lymphocytic background, with single, scattered tumor epithelial cells. (B) CK immunohistochemistry 200×. Scattered epithelial cells. (C) CD20 immunohistochemistry 200×. Partial positive tumor cells. (D) TdT immunohistochemistry 200×. Partial positive tumor cells. (E) CD4 immunohistochemistry 200×. Tumor cells containing a large number of mature T lymphocytes.

After the patient was transferred to our hospital, a complete blood cell count indicated WBC of 2.1 × 10^9^/L, NE 0.0 × 10^9^/L, hemoglobin 94 g/L, and platelet 328 × 10^9^/L. Chest computed tomography scan revealed a 7.6 × 6.3 cm anterior mediastinum with uneven density, smooth borders, and a significantly enhanced mass in the arterial phase after enhancement (Fig. 1). Bone marrow analysis and biopsy showed that the proportion of granulocytes at each stage decreased or was absent (Fig. 2). Flow cytometric analysis of the bone marrow demonstrated a significant reduction in the number of granulocytes (Fig. 3). In addition to the 3 abovementioned typical abnormalities, the patient had antinuclear antibodies, autoimmune thyroiditis, and Epstein–Barr virus DNA positivity. However, the immunoglobulin levels were normal, and peripheral blood flow cytometry showed a CD4:CD8 ratio of 0.98 (Table 1). The patient had a recurrent fever and persistent granulocyte count of 0. She was sequentially treated with cefoperazone–sulbactam, imipenem, vancomycin, piperacillin–tazobactam, and voriconazole for infection control. Additionally, human G-CSF at a dose of 300–450 μg/d was administered to enhance granulocytes production. Unfortunately, the patient did not experience relief from the fever or agranulocytosis despite these interventions. Mediastinal tumor resection was performed under sterile conditions after 3 weeks of agranulocytosis, and a biopsy of the mass was performed with the anatomopathological result of a type B1 thymoma. For the examination of thymoma tissues, the samples were embedded in paraffin and sectioned. The sections were then dewaxed and rehydrated. The resultant cells tested positive for CD79a (scattered), CD20, Ki67 (50%), CD58 (scattered), CD4, CD8, CK, and TdT but negative for CD10 and CD35 (Fig. 4). Postoperatively, the patient developed persistent agranulocytosis. On the 11th day after the operation, the patient’s treatment regimen was supplemented with CSA capsules at a dosage of 100 mg every 12 hours. After 9 days, the patient’s granulocyte count began to increase, reaching a value of NE 0.4 × 10^9^/L. By day 11, the granulocyte count had returned to normal, reaching NE 4.6 × 10^9^/L. However, when treated with CSA, the patient experienced repeated nausea and vomiting, and the antiemetic drugs were ineffective. After the granulocyte levels returned to normal, the patient refused to continue taking cyclosporine.

**Table 1:** Laboratory data.

	Admission	Day 44 (22 d after surgery)	Day 93 (relapse)	Reference values
Complete blood count				
WBC	1.90	17.80	3.60	3.5–9.5 (×10^9^/L)
NE	<0.01	4.60	0.80	1.8–6.3 (×10^9^/L)
LY	1.80	6.20	2.50	1.1–3.2 (×10^9^/L)
MO	0.10	0.40	0.30	0.1–0.6 (×10^9^/L)
EOS	0.01	0.18	<0.01	0.02–0.52 (×10^9^/L)
BAS	<0.01	0.18 × 10^9^/L	<0.01	0.0–0.06 (×10^9^/L)
HGB	102	103	117	115–150 (g/L)
PLT	302	253	259	125–350 (×10^9^/L)
Thyroid function and antibody				
TSH	0.117	3.366	0.372	0.35–4.94 (μIU/mL)
TG-Ab	>1000.00	826.61	597.82	0–4.11 (IU/mL)
TPO-Ab	>1000.00	964.70	>1000.00	0–5.61 (IU/mL)
Immune markers				
IGG	13.90	—	15.70	7.51–15.6 (g/L)
IGA	1.94	—	2.48	0.82–4.53 (g/L)
IGM	1.15	—	3.17	0.46–3.04 (g/L)
ANA	1:1000	1:320	1:320	Negative
Sm	Negative	Negative	Weak positive	Negative
CD4+/CD8+	0.98	0.76	0.85	0.8–3.2
Evaluation of T-cell clonality	Negative	—	—	Negative
Virus				
EB-DNA	8.23 × 10^3^ copies/mL	—	below the limit of detection	<400

ANA = antinuclear antibody, BAS = basophil, EB = Epstein–Barr virus, EOS = eosinophil, HGB = hemoglobin, IGA = immunoglobulin A, IGG = immunoglobulin G, IGM = immunoglobulin M, LY = lymphocyte, NE = neutrophil, PLT = platelet, Sm = anti-Sm antibody, TG-Ab = thyroglobulin antibody, TPO-Ab = thyroid peroxidase antibody, TSH = thyroid-stimulating hormone, WBC = white blood cell.

**Figure 5.**
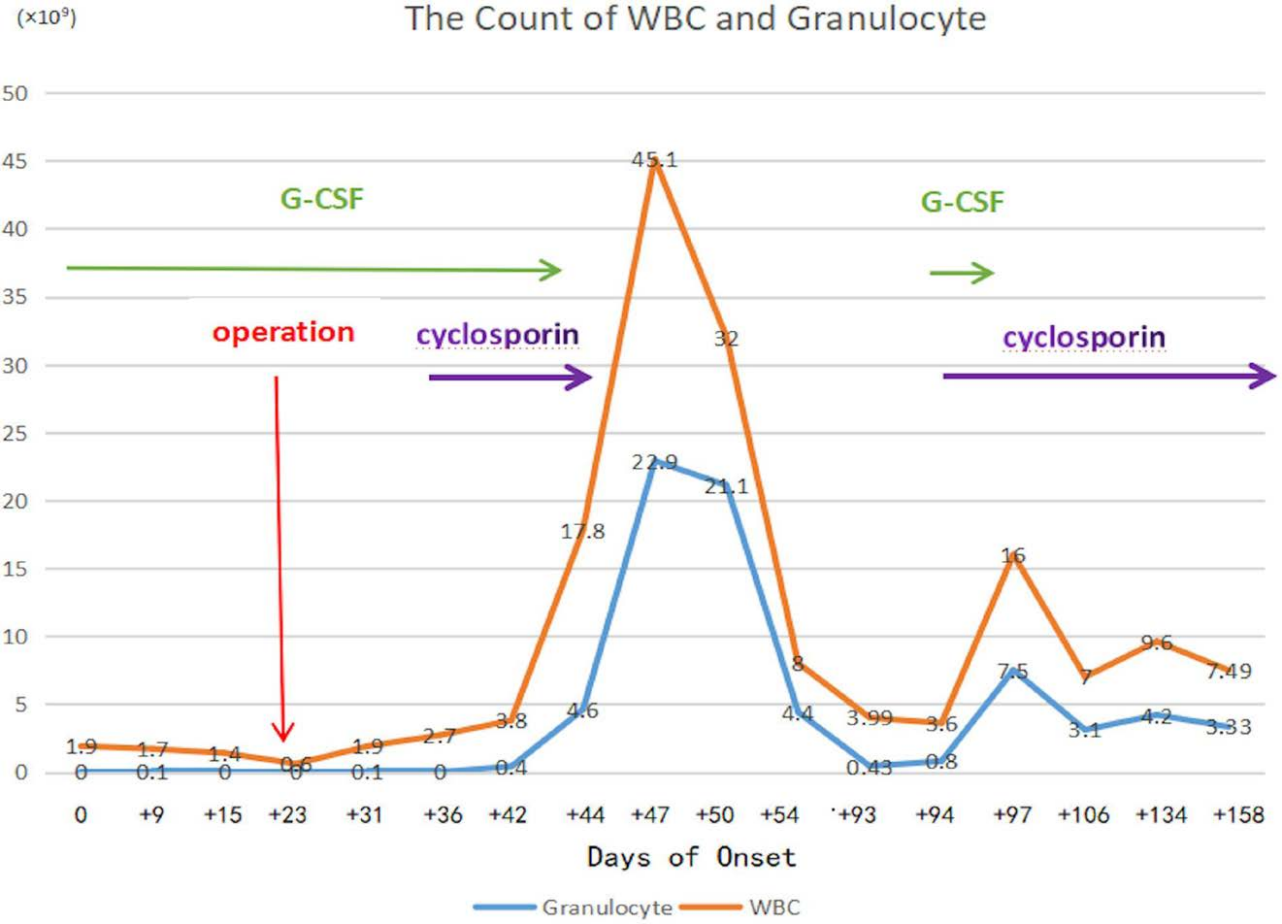
Trends in white blood cell and neutrophil counts following treatment in our patient.

The results of the subsequent outpatient monitoring of granulocytes were normal. One and a half months later, the patient experienced a relapse of agranulocytosis, and the granulocyte count was measured as 0.43 × 10^9^/L. Cyclosporine A was reintroduced at a dose of 75 mg every 12 hours in combination with G-CSF. Thereafter, the patient continued to receive CSA, and her granulocyte levels remained within the normal range up until the present time (Fig. 5).

## 3. Discussion

Paraneoplastic syndromes in patients with thymomas include myasthenia gravis, pure erythrocyte aplastic anemia, hypogammaglobulinemia (Good syndrome), and PWCA. PWCA with thymoma is a rare disease (1% of all thymomas).^[8]^ The patient in this study did not show any symptoms or laboratory findings indicative of myasthenia gravis, and there was no evidence of hypogammaglobulinemia reported. The blood test results revealed decreased or absent granulocyte levels and increased levels of erythroid and megakaryocyte components, as observed in the PWCA, marrow analysis, and biopsy. The patient in this study had a definite diagnosis of thymoma complicated by PWCA. A review of case reports over the past 3 decades was conducted (Table 2). Similarities to the cases presented in this study were observed. Among the 16 rare cases, 8 had hypogammaglobulinemia (4 with Good syndrome); 2 myasthenia gravis; 2 autoimmune thyroiditis; 1 coagulation factor XI deficiency; 1 pure red cell aplastic anemia; and 1 immune thrombocytopenia. The etiology of thymoma combined with PWCA is still challenging to elucidate in existing research; the current consensus is that PWCA is still autoimmune origin.^[20]^

**Table 2:** Pure white cell aplasia associated to thymoma reported in literature.

No	Reference	Age/sex	Bone marrow	Timing of onset PWCA	Comorbid diseases	PRCA treatment	Outcome	Thymoma histology	Laboratory indicators	Granulocyte status before and after treatment
1	Ackland et al^[9]^	70/M	Myelopoiesis absent	7 yr after thymectomy	Myasthenia gravis Hypogammaglobulinemia	IVIg	Died of sepsis	Spindle cell	Antigranulocyte antibodies(+)	0.1 × 10^9^/L Agranulocytosis after treatment
2	Mathieson et al^[10]^	36/F	Myelopoiesis absent	10 yr after thymectomy	Myasthenia gravis	Plasmapheresis Azathioprine Prednisolone	Survived	Lymphoepithelial type	Anti-AChR(+) Anti-MUSK(+)	0.8 × 10^9^/L 3.6 × 10^9^/L
3	Postiglione et al^[11]^	68/F	Myelopoiesis absent	Concomitant	Deep venous thrombosis PRCA	Prednisone IVIG G-CSF Plasmapheresis Cyclophosphamide	Died of fungal sepsis	Spindle cell	ANA(+) Low IGG	0.6 × 10^9^/L 41.9 × 10^9^/L
4	Yip et al^[12]^	51/M	Promyelocyte arrest	Concomitant	/	Prednisone G-CSF	Survived	Spindle cell	Anti-MUSK(+)	0.4 × 10^9^/L 25.4 × 10^9^/L
5	Yip et al^[12]^	52/F	Myelopoiesis absent	Concomitant	Lethargy Weight loss	GM-CSF IVIg Methylprednisolone Plasmapheresis Thymectomy Cyclophosphamide	Died of sepsis	Spindle cell	ANA(+)	0.09 × 10^9^/L Agranulocytosis after treatment
6	Crawford et al^[13]^	59/M	Myelopoiesis absent	Concomitant	Hypogammaglobulinemia Good’s syndrome	Methylprednisolone Prednisone Azathioprine	Survived	Spindle cell	Low IGG, IGA, and IGM CD4/CD8 ratio reversed	0.1 × 10^9^/L 11.5 × 10^9^/L
7	Fumeaux et al^[14]^	76/F	Myelopoiesis absent	Concomitant	Autoimmune thyroiditis Type 1 diabetes	IVIg G-CSF Thymectomy Cyclosporine A Prednisone	Survived	Malignant cortical thymoma (type B2)	Islet cell Antibodies(+) Thyroperoxidase thyroglobulin antibodies(+) Anti-MUSK(+)(+)	0 × 10^9^/L 20 × 10^9^/L
8	Alvares et al^[15]^	59/M	Myelopoiesis absent Relapse Promyelocyte arrest	Concomitant	/	G-CSF Thymectomy Plasmapheresis Campath-1H Cyclosporine A Mycophenolate mofetil	Survived	Spindle cell thymoma (type A)	AChR(+) ANA(±)	0.1 × 10^9^/L Normal after treatment
9	Jethava et al^[16]^	45/M	Myelopoiesis absent	Concomitant	Factor XI deficiency	IVIg G-CSF Cyclosporine A Thymectomy	Survived	Type AB thymoma	APTT prolonged Low IGG	0.0 × 10^9^/L 1.2 × 10^9^/L
10	Akinosoglou et al^[17]^	70/F	Block of myeloid maturation	Concomitant	Good’s syndrome Cryptococcus infection	G-CSF IVIg Dexamethasone	Survived	Type A thymoma	CD4/CD8 ratio reversed γ-globulin fraction reduced Low IgA and IgM	0.2 × 10^9^/L 5.7 × 10^9^/L
11	Okusu et al^[18]^	72/M	Hypoplasia of only leukocyte progenitor cells	Concomitant	Good’s syndrome Myocarditis	IVIg	Died of sepsis	Type B2 thymoma	Low IgG, IgA, and IgM	0.0 × 10^9^/L Agranulocytosis after treatment
12	Kobayashi et al^[2]^	63/M	Myelopoiesis absent	Concomitant	Hypogammaglobulinemia	G-CSF Cyclosporine A Thymectomy	Survived	Type A thymoma	Monoclonal rearrangements of T-cell receptors (−)	0.0 × 10^9^/L 2.5 × 10^9^/L
13	Uy et al^[19]^	65/F	Myelopoiesis absent	Concomitant	Good’s syndrome Dermatitis	IVIg G-CSF Thymectomy Cyclosporine A	Survived	Type A + B2 thymoma	Low serum immunoglobulin	0.0 × 10^9^/L 2.8 × 10^9^/L
14	Céspedes López et al^[20]^	33/M	Myelopoiesis absent Promyelocyte maturational arrest	Concomitant	Gangrenous ecthyma	IVIg CSF Cyclosporine A Thymectomy	Survived	Mixed type AB thymoma	Low IgG and IgA CD4+/CD8+ ratio inversion	0.0 × 10^9^/L 17 × 10^9^/L
15	Youssef et al^[21]^	74/M	Granulocytic hypoplasia	2 wk after thymectomy	Immune thrombocytopenia	IVIg G-CSF Cyclosporine A Prednisone	Died from sepsis and multiorgan failure	Type B1 thymoma	Low platelets	0.1 × 10^9^/L 8.0 × 10^9^/L
16	Case	68/F	Myelopoiesis absent	Concomitant	Hashimoto thyroiditis	IVIg G-CSF Cyclosporine A	Survived	Type B1 thymoma	Thyroperoxidase thyroglobulin antibodies(+)	0.1 × 10^9^/L 4.6 × 10^9^/L

/ indicates similarities to the cases presented in this study were observed.

ANA = antinuclear antibody, Anti-AChR = anti-acetylcholine antibody, Anti-MUSK = anti-smooth muscle antibody, F = female, G-CSF = granulocyte colony-stimulating factor, IVIG = intravenous immunoglobulin G, M = male.

PWCA is a relatively rare disorder with only a few reported cases. Currently, there are numerous causes of PWCA (e.g., autoimmunity, drugs, viral infections, and thymoma). More specifically, several cases of thymomas combined with PWCA have been reported. Possible causes of this disease are as follows. The first is antibody-mediated autoimmunity, which suppresses granulocyte production. Inhibitors of colony-forming unit granulocytes and macrophages (CFU-GMs) have been implicated in the pathogenesis of PWCA. The CFU-GM assay uses normal human bone marrow cells incubated with patient or control serum under standard conditions. The serum of patients with PWCA and thymomas contains inhibitory factors for CFU-GM.^[2,9]^ The second involves the cytostatic mechanism of T lymphocytes. The thymus is an essential organ for T-lymphocyte maturation. T lymphocytes play a certain role in the production of autoantibodies against granulocyte-producing cells.^[20]^ In the immature environment of T cells in thymoma, T cells cannot tolerate and mature in the thymus, and autoreactive T cells are activated.^[22]^ The escape of autoreactive T lymphocytes can activate B cells to initiate an autoimmune response that inhibits myeloid precursor cells.^[23]^ In our case, peripheral and bone marrow lymphocytes were dominated by T lymphocytes, whereas an abnormal immunophenotype was absent. Both T- and B-cell clonality evaluations were negative in 16 patients, including our case (Table 2). Only 3 patients had a CD4/CD8 ratio reversed in T cells. Two patients had a history of Hashimoto thyroiditis, and the other patients with thymoma had agranulocytosis in the absence of an immune system disease. These findings indicate potential factors that inhibit the growth of myeloid progenitors.

Different therapeutic management strategies have been developed for patients with PWCA and thymomas. The treatment of 16 patients was studied (Table 3). First-line treatment options include glucocorticoids, G-CSF, and plasmapheresis; however, these treatments are often ineffective. Thymectomy has little effect on the survival of patients with this disease. In general, surgical removal of the thymoma is recommended to rule out its possible role in the autoimmune process. Of the 16 patients, 3 had PWCA after thymoma surgery, and in 3 patients with PWCA correlated with thymoma, PWCA recurrence was observed after thymectomy. The possibilities that thymoma directly causes PWCA and that thymectomy can cure PWCA were denied. Management of agranulocytosis leading to infection is of great significance throughout the course of the disease. Five of the 16 patients died of sepsis. The patient in this study did not develop fatal infections for up to 46 days of agranulocytosis, primarily because of the use of laminar flow beds. Thymectomy was performed in 7 patients with agranulocytosis, which is a critical issue in managing postoperative agranulocytic infection. Although thymectomy does not necessarily reverse PWCA, the patients’ granulocyte levels did not recover even after undergoing thymectomy. However, the bone marrow did respond to the addition of other drugs.

**Table 3:** Treatment options and number of effective cases in literature.

First-line treatment options	Remission cases/total cases	Second-line treatment options	Remission cases/total cases	Relapse treatment options	Remission cases/total cases
IVIg	0/2	Thymectomy + cyclosporine A + G-CSF	3/3	G-CSF + cyclosporine A + prednisone	2/2
IVIg + G-CSF	0/3	Thymectomy + methylprednisolone	1/1	Mycophenolate mofetil + cyclosporine A	1/1
IVIg + glucocorticoid	1/1	Thymectomy + Campath-1H + cyclosporine A	1/1		
Glucocorticoid + azathioprine	1/1	Thymectomy + IVIg + glucocorticoid + plasmapheresis + cyclophosphamide	0/1		
G-CSF + cyclosporine A	2/2	Glucocorticoid + azathioprine	1/1		
G-CSF + plasmapheresis	0/1	Thymectomy + plasmapheresis + cyclophosphamide A	1/1		
G-CSF/IVIg + glucocorticoid	1/5	IVIg + G-CSF + cyclophosphamide A	1/1		
Plasmapheresis + glucocorticoid	0/1				

G-CSF = granulocyte colony-stimulating factor, IVIG = intravenous immunoglobulin G.

The use of azathioprine, CSA, or cyclophosphamide is more effective in treating thymoma-associated PWCA with immunosuppressive drugs, regardless of first-line to second-line or relapsed therapy. Seven of these cases were effectively treated with CSA (Table 3), and the patient in this study benefited from CSA treatment both after surgery and after recurrence. These results indicate that low-dose CSA after PCWA therapy was sufficient to maintain long-term remission after thymectomy.^[15]^ Accordingly, patients with thymoma-associated PCWA benefit more from first-line immunosuppressive therapy. Interestingly, or therapeutic efficacy, the etiology of patients with thymoma-related PCWA may be that the effector function of mature T cells in an abnormal thymic environment is a major factor in the disease.

## 4. Limitations

This study has certain limitations. First, due to the disease’s rarity, it is hypothesized that the mechanism of thymoma-associated PWCA involves T-cell activity. Considering the thymus’s role in T-lymphocyte maturation and the ability of T lymphocytes to produce autoantibodies against granulocytes, the focus was on the repeated administration of CSA, which suppresses T lymphocytes. Second, the literature review was limited to patients with thymoma and granulocyte deficiency, excluding those with leukopenia. Nonetheless, further research is warranted given the limited number of cases.

## 5. Conclusions

The pathogenesis of thymomas in patients with PWCA is closely linked to T-cell activation. Our case study and literature review suggest that thymectomy alone is insufficient for curing this rare disease. The immunosuppressive agent CSA can eliminate activated T cells; however, short-term administration proves ineffective, necessitating a longer period of treatment. After surgery, our patient continued to take CSA orally, and WBCs returned to normal levels. Owing to the rare nature of this case, we explored its unique characteristics and documented our treatment experience for this particular case.

## Author contributions

**Writing** – **review & editing:** Yang Yang.

**Data curation:** Chunmei Chen, Bingrong Zheng.

**Methodology:** Chunmei Chen, Xiajun Chen.

**Formal analysis:** Bingrong Zheng.

**Resources:** Liping Fan.

**Validation:** Liping Fan.

**Investigation:** Xiajun Chen.

**Conceptualization:** Meiwei Hu.

**Writing – original draft:** Meiwei Hu.
